# [Cost]effectiveness of withdrawal of fall-risk increasing drugs versus conservative treatment in older fallers: design of a multicenter randomized controlled trial (IMPROveFALL-study)

**DOI:** 10.1186/1471-2318-11-48

**Published:** 2011-08-21

**Authors:** Klaas A Hartholt, Nicole DA Boyé, Nathalie Van der Velde, Esther MM Van Lieshout, Suzanne Polinder, Oscar J De Vries, Albert JH Kerver, Gijsbertus Ziere, Milko MM Bruijninckx, Mark R De Vries, Francesco US Mattace-Raso, André G Uitterlinden, Ed F Van Beeck, Paul Lips, Peter Patka, Tischa JM Van der Cammen

**Affiliations:** 1 Department of Internal Medicine - Section Geriatric Medicine, Erasmus MC, University Medical Rotterdam, P.O. Box 2040, 3000 CA Rotterdam, The Netherlands; 2 Department of Surgery-Traumatology, Erasmus MC, University Medical Center Rotterdam, P.O. Box 2040, 3000 CA Rotterdam, The Netherlands; 3 Department of Public Health, Erasmus MC, University Medical Center Rotterdam, P.O. Box 2040, 3000 CA Rotterdam, The Netherlands; 4 Department of Internal Medicine, VU university medical center, P.O. Box 7057, 1007 MB Amsterdam, The Netherlands; 5 Department of Surgery-Traumatology, Sint Franciscus Gasthuis, Kleiweg 5003045 PM Rotterdam, The Netherlands; 6 Department of Geriatric Medicine, Harbour Hospital Rotterdam, Haringvliet 23011 TD, Rotterdam, The Netherlands; 7 Department of Surgery-Traumatology, IJsselland Hospital, P.O. Box 690, 2900 AR Capelle a/d IJssel, The Netherlands; 8 Department of Surgery-Traumatology, Reinier de Graaf Groep, P.O. Box 5011, 2600 GA Delft, The Netherlands; 9 Department of Internal Medicine, Erasmus MC, University Medical Rotterdam, P.O. Box 2040, 3000 CA Rotterdam, The Netherlands

## Abstract

**Background**

Fall incidents represent an increasing public health problem in aging societies worldwide. A major risk factor for falls is the use of fall-risk increasing drugs. The primary aim of the study is to compare the effect of a structured medication assessment including the withdrawal of fall-risk increasing drugs on the number of new falls versus 'care as usual' in older adults presenting at the Emergency Department after a fall.

**Methods/Design**

A prospective, multi-center, randomized controlled trial will be conducted in hospitals in the Netherlands. Persons aged ≥65 years who visit the Emergency Department due to a fall are invited to participate in this trial. All patients receive a full geriatric assessment at the research outpatient clinic. Patients are randomized between a structured medication assessment including withdrawal of fall-risk increasing drugs and 'care as usual'. A 3-monthly falls calendar is used for assessing the number of falls, fallers and associated injuries over a one-year follow-up period. Measurements will be at three, six, nine, and twelve months and include functional outcome, healthcare consumption, socio-demographic characteristics, and clinical information. After twelve months a second visit to the research outpatient clinic will be performed, and adherence to the new medication regimen in the intervention group will be measured. The primary outcome will be the incidence of new falls. Secondary outcome measurements are possible health effects of medication withdrawal, health-related quality of life (Short Form-12 and EuroQol-5D), costs, and cost-effectiveness of the intervention. Data will be analyzed using an intention-to-treat analysis.

**Discussion**

The successful completion of this trial will provide evidence on the effectiveness of withdrawal of fall-risk increasing drugs in older patients as a method for falls reduction.

**Trial Registration**

The trial is registered in the Netherlands Trial Register (NTR1593)

## Background

Falls constitute one of the most common and serious public health problems in older populations. Fall incidents are associated with considerable morbidity and mortality [1-3]. Even a low energetic trauma, such as an unintended fall, can lead to major injuries in older adults with long-term consequences [4,5]. The incidence of falls and the severity of fall-related complications rises steeply beyond the age of 65 years [1,2,4-6]. Approximately 72,000 older adults visit an Emergency Department in the Netherlands each year due to a fall. Over 30,000 are hospitalized, and nearly 1,600 elderly die due to a fall per year [7,8]. The large burden of fall-related healthcare consumption is leading to high healthcare costs in western societies [5,9,10]. Over the past decades several risk factors for falls have been identified. Major risk factors include one or more previous falls, mobility impairments, high age, and the use of fall-risk increasing drugs [11,12]. The majority (73%) of older persons use one or more drugs [13]. In 2008, nearly half of all drug prescriptions in the Netherlands were delivered to persons aged 65 years and older who constituted only 15% of the Dutch population in that year [14]. Adverse Drug Reactions are frequently seen in older adults [15]. A meta-analysis of observational studies showed an increased fall risk with certain drug groups, i.e., psychotropic [16] and cardiovascular drugs [17]. Approximately three-quarters of the community dwelling elderly used at least one prescribed drug, and about a third used at least one fall-risk increasing drug [13].

There is evidence that withdrawal, reduction, or substitution of fall-risk increasing drugs can reduce fall risk in older adults. Only one small, randomized controlled trial on drug withdrawal has been performed [18]. Campbell *et al. *found that withdrawal of psychotropic medication significantly reduced the risk of falling, but permanent withdrawal proved very difficult to achieve. Therefore the authors made recommendations for a larger randomized controlled trial (RCT) to study the single effect of drugs assessment and drugs modification on fall risk. A recent prospective cohort study with a two-month follow-up period showed that the withdrawal of fall-risk increasing drugs was associated with a reduction in falls [19].

Furthermore, an increased susceptibility to certain adverse drug reactions may partly be due to genetic polymorphisms that alter responses of individual persons to various drugs [13]. A possible cause might be the pathway of hepatic drug metabolization by the cytochrome P-450 family of biotransformation enzymes [20]. Consequently, poor, extensive and ultra-rapid metabolizers for certain cytochrome pathways and membrane bound transporters can be distinguished [21], which influence the pharmacodynamics and pharmacokinetics. The majority of fall-risk increasing drugs are metabolized by a small number of enzymes, the major ones being CYP450 2D6, 2C9, 2C19 and 3A4/5 [22]. Due to polypharmacy among older adults, the risk of a CYP 450 interaction increases.

A systematic fall-related drugs assessment combined with medication changes and a one-year follow-up assessment among older fallers may contribute to a reduction in the incidence of new falls and related consequences [19]. At this moment a structured medication assessments are not part of standard care of older fallers presenting at the Emergency Department. In the Netherlands, the current care of fall-related injuries consists of treatment of the injuries of the fall. However, before a systemic fall-related medication assessment can be incorporated in the routine work-up of older persons presenting with a fall, further evidence is required. The aim of this randomized controlled trial is to compare the effect of withdrawal of fall-risk increasing drugs versus 'care as usual' on future falls. The primary outcome of this study is be the number of new falls and fallers. Secondary outcome measurements are possible health effects of medication withdrawal, health-related quality of life, costs, and cost-effectiveness of the intervention.

## Methods/Design

**Figure 1:**
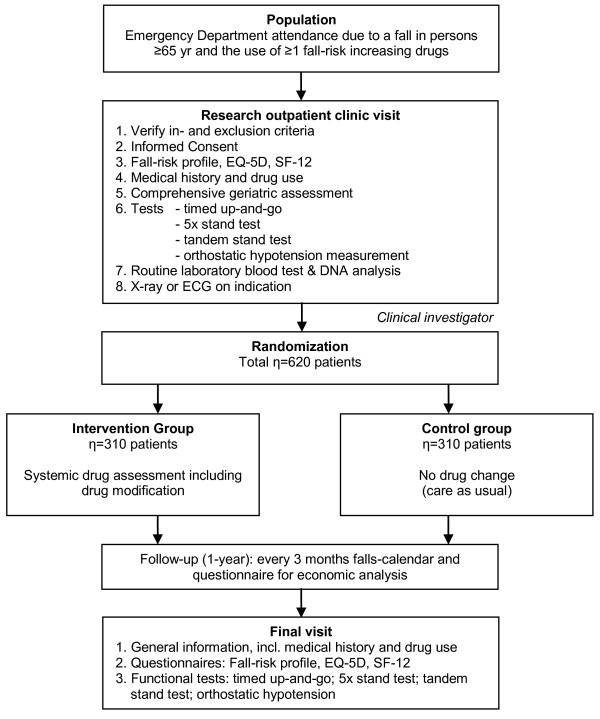
**Flow chart**.

The study is designed as a multicenter RCT with a one-year follow-up period in the Netherlands. The Medical Ethics review board of the Erasmus MC, University Medical Center, approved the study protocol. The study started in October 2008. The flow chart is shown in Figure 1.

### Study population

Patients aged 65 years and over, who visit the Emergency Department of a participating hospital due to a fall, are eligible for inclusion. A fall is defined as coming to rest unintentionally on the ground or a lower level with or without losing consciousness, but not induced by acute medical conditions, *e.g*., stroke, or exogenous factors such as a traffic accident [23].

Patients meeting the following inclusion criteria are eligible for enrollment:

1. Aged 65 years or older (no upper age limit)

2. Attended the Emergency Department due to a fall incident

3. Taking one or more fall-risk increasing drugs for at least two weeks prior to the fall

4. Mini-Mental State Examination score of 21/30 points or over

5. Able to walk independently

6. Community dwelling

7. Provision of informed consent by patient

If any of the following criteria applies, patients will be excluded:

1. Patient participation in another trial

2. Fall not meeting criteria of specified definition

3. Likely problems, in the judgment of the investigators, with maintaining follow-up (e.g., patients with no fixed address)

4. Not willing to complete the research protocol (such as attending at a follow-up visit)

### Procedure

All persons visiting the Emergency Department due to a fall receive care as usual for their injuries. Within two weeks following the Emergency Department attendance, patients are contacted by telephone with information about the study. All eligible study participants will receive written information about the study and all interested patients will receive an appointment for the research outpatient clinic. The visits to the outpatient clinic take place within two months after Emergency Department attendance. If the patient meets all eligibility criteria and no exclusion criteria are present at the research outpatient clinic, the patient will be asked to sign the Informed Consent Form before the study procedures take place. Patients who do not meet the inclusion criteria will be excluded. During the outpatient clinic visit a record is made of the falls risk profile (FRP), falls history, health-related quality of life (HRQoL) and physical performance are measured of all study participants. Furthermore, a geriatric assessment and a standardized medication assessment will take place. Eligible patients will be randomized to one of the treatment arms, the intervention group versus 'care as usual'. The aim in the intervention group will be to reduce fall-risk increasing drugs, and in the 'care as usual' group no (medication) change will be made. All included participants receive a Falls Calendar for reporting falls during a one-year follow-up period as well as a cost-evaluation form at three, six, nine and twelve months after the first research outpatient clinic visit. One year after the first visit, the study participants are invited for a final visit to the research outpatient clinic in order to reassess the FRP, falls history, HRQoL, and physical performance. Adherence to their medication is also evaluated. After the final visit to the outpatient clinic a brief letter concerning the study start and completion will be sent to the patient's General Practitioner. Table 1 shows the schedule of events of this study.

**Table 1:** Schedule of events

	Screening	1^st ^visit	3 months	6 months	9 months	12 months
Telephone call	X					
Information package	X					
Informed Consent		X				
Randomization		X				
Baseline data		X				
EQ-5D		X				X
SF-12		X				X
FRP		X				X
Orthostatic hypotension test		X				X
Complications			X	X	X	X
Falls calendar			X	X	X	X
Healthcare consumption			X	X	X	X
ADL		X				X
Physical functioning (VAS)		X				X

EQ-5D, EuroQol 5-D questionnaire; SF-12, Short Form-12; FRP, Fall Risk Profile; ADL, Activities of Daily living; VAS, Visual Analogue Scale.

### Randomization

Participants will be allocated to one of two treatment arms using a web-based randomization program that will be available 24 hours a day. Variable block randomization will be accomplished via a trial website. Allocation will be random. It is not possible to blind the geriatrician and patients for the allocation of the study group.

### Intervention

The single intervention will consist of a systematic fall-related medication assessment combined with drug withdrawal or modification, if safely possible. Fall-risk increasing drugs, as defined in the literature [16,17,19,24], will be stopped, reduced or substituted with potentially safer drugs in the intervention group. A complete list of fall-risk increasing drugs, based on current literature, is shown in Table 2.

**Table 2:** Drugs classified as fall-risk increasing drugs in the IMPROveFALL study

Category	Drug type
Central nervous system	**anxiolytics/hypnotics **(benzodiazepines and others); **antidepressants (**tricyclic antidepressants, selective serotonin reuptake inhibitors, serotonin-norepinephrine reuptake inhibitors and monoamine oxidase inhibitors), **neuroleptics **(dopamine D2-receptor agonists and serotonin dopamine receptor antagonists)

Cardiovascular	**Antihypertensives **(diuretics, beta-adrenoceptor blockers, alpha-adrenoceptor blockers, centrally acting antihypertensives, calcium channel blockers, angiotensin converting enzyme inhibitors and angiotensin receptor blockers); **Anti-arrhythmic drugs **(Antiarrhythmics, nitrates, digoxin, vasodilators)

Anti-inflammation	NSAIDs

Gastro-Intestinal	Antacids (H-2 receptor antagonists)

Analgesics	Opioids

Pulmonary	Sympathomimetics, anti-histaminics

Diuretics	Thiazide diuretics, loop diuretics

For each drug, the clinical investigator will assess whether the initial indication still exists. Proposed changes in medication will be discussed with a senior geriatrician and the participant's General Practitioner and with the prescribing doctor if other than the General Practitioner. If consensus is obtained, fall-risk increasing drugs will be discontinued when considered redundant, reduced in dose over a one-month period, if safely possible, or substituted for potentially safer drugs if necessarily and available. For each drug modification, the clinical investigator will follow the standardized instructions of the Dutch National Formulary [25], and a clinical pharmacologist will be available for advice when needed. A research nurse will offer counseling and evaluate possible negative effects by weekly telephone calls over a period of 1 month, and discuss any problems with the clinical investigator and the geriatrician (project leader).

### Outcome measures

The primary outcome measure will be the incidence of new falls, fallers, based on the Falls Calendar. Secondary outcome measures will be fall-related injuries, generic health-related HRQoL, compliance, quality adjusted life years (QALY), genetic polymorphisms associated with increased adverse drug reactions, and positive or negative health effects, cost, and cost-effectiveness.

### Measurements

### Medication use

Medication use will be assessed by registering the drug names directly from the medication packaging. For each drug, both prescription and over-the-counter (OTC), the name, intake frequency, dosage, start and stop dates, and whether the drug was prescribed before or after the fall will be registered. The information will be verified and compared with data retrieved from the patients' General Practitioner and local pharmacist.

### Quality of life

The level of independency of the activities of daily living (ADL) will be examined using the Barthel Index (ranging from zero for full independency to 20 for full dependency) [26]. Quality of life will be measured using the Dutch version of the SF-12 and EQ-5D (EuroQol) questionnaire. The EQ-5D has been designed by the Euro-HRQoL Group to assess the experienced general quality of life in large populations in order to provide a simple, generic measure of health for clinical and economic appraisal [27]. The EQ-5D questionnaire covers five health domains (mobility, self-care, usual activities, pain/discomfort, and anxiety/depression) and a Visual Analogue Scale (VAS) to record the current experienced health status. The EuroQol (EQ-5D) is a validated and extensively used general health questionnaire to measure quality of life [28,29]. It is recommended for the assessment of HRQoL in trauma patients, especially for economic assessments [30]. The SF-12 contains 12 questions and has been designed and validated to assess the quality of life in large population studies [31,32]. Fall-risk will be assessed using a validated FRP [33]. The FRP contains five questions, two measurements (handgrip strength and body weight), and two interacting items. Hand grip strength will be measured using a digital strain-gauged dynamometer (Takei TKK 5401, Takei Scientific Instruments Co, Ltd., Tokyo, Japan). Body weight will be measured with a calibrated beam scale. For each item points are scored and summed (range 0-30), where zero represents a low risk of recurrent falling and 11 and over indicates a high risk of recurrent falling (2 or more falls in the next 12 months) [33].

### Physical performance

In order to assess the physical activity, three tests will be conducted. First, the chair stand test, which is a standardized test in which the participant stands up and sits down five consecutive times. The patient is not permitted to use the chair's arms supports during standing up or sitting down [34]. The Timed Up-and-Go test (TUG-test) will be conducted, in which the participant has to stand up from sitting position and walks three meters along a line, perform a 180 degree turn and walk back to the chair and sit down [34]. A tandem stand test will be used in order to assess balance. The test will be performed in standing position, in which the patient has to stand fully independently for 10 seconds with both feet in front of each other, and is scored as correct or failed. All three mobility tests are conducted twice, and the best time (where appropriate) will be used.

Orthostatic hypotension will be measured by using a calibrated sphygmomanometer, in supine position followed by five minutes standing straight up. The blood pressure will be measured in supine position and after one, two, three, four, and five minutes standing. The blood pressure is registered in millimetres of mercury (mmHg), heart rate in beats per minute. Orthostatic hypotension is defined as a decrease of 20 mmHg systolic or a decrease of 10 mmHg diastolic in standing position [35].

### Costs

The total direct and indirect costs of both fall-risk increasing drugs withdrawal and 'care as usual' will be measured. All analysis will be performed in accordance with Dutch guidelines for economic evaluations [36]. Direct healthcare costs include the additional costs of the systematic fall-related drugs assessment and modification, drug consumption (including the costs for substitution drugs), and fall-related and non-fall-related healthcare consumption during one year of follow-up (e.g. General Practitioner, outpatient visits, and hospital admissions).

Real medical costs are calculated by multiplying the volumes of health care use with the corresponding unit prices. For the intervention (systematic fall-related drugs assessment) the full cost price will be calculated and for the other health care costs standard cost prices will be used as published by Oostenbrink [36]. The full cost price of patient identification at the Emergency Department and the systematic fall-related drugs assessment will be determined based upon time measurements and employment of personnel. Costs of medication use will be recorded in the study, and unit costs will be determined with information from the National Dutch Formulary [25].

Healthcare consumption, both fall and non-fall related, and patient costs will be recorded from the Hospital Information System for hospital care, and three-monthly questionnaires for other healthcare and patient costs. These will be supplemented with data on healthcare costs of injury from previous research [9]. The number of injuries prevented will be calculated with data recorded in the study, supplemented with epidemiological data on falls and injury risks.

Cost-effectiveness will be assessed by calculating the incremental cost-effectiveness ratio, defined here as the difference in average costs between medication assessments including withdrawal of fall-risk increasing drugs and 'care as usual' and by the difference in prevented fall-related injury. Secondary, a cost-utility analysis will be performed, *i.e*., as cost per Quality Adjusted Life Years (QALY). Policy makers and health economists have proposed that costs varying from €25,000 up to €75,000 per QALY may be considered as acceptable [37,38].

The QALY combines mortality and morbidity into a single number. The morbidity component is referred to as HRQoL and is based on a descriptive health-state measure. Because of a long track record in health economic analyses, the EQ-5D measure will be used for this purpose [28]. Furthermore, the lifetime health effects (cardiovascular events such as myocardial infarction, stroke, and mortality) due to possible increased cardiovascular risks (*i.e*., cardiac failure, rebound hypertension) will be calculated with existing models for cardiovascular disease risk management. In accordance with guidelines for differential discounting, effects will be discounted at a rate of 1.5% and costs at 4% per year [39].

Full blood for DNA isolation will be drawn during the first visit (5 mL). The blood will be stored at -80 degrees Celsius, until DNA-isolation will take place. After DNA isolation, polymorphisms (CYP1A2, CYP2C9, CYP2C19, CYP2D6, CYP2E1 en CYP3A4) will be analyzed using the TaqMan allelic discrimination assays on the ABI Prism 9700 HT sequence detection system. If needed, other polymorphisms will be added to the analysis.

### Follow-up

Patients will be followed for one year. After the first visit to the research outpatient clinic patients receive a Falls Calendar [33]. During a one-year follow-up period, the participant will be asked to record every week whether they have experienced a fall that week. The 3-monthly calendar sheet will be returned once per 3-months by mail. Cost-effectiveness will be measured using a cost-evaluation questionnaire. Participant can register the number of visits to physicians, therapists, day care centers, hospitalizations, adaptations of the living area, and the current living location (*e.g*., home or nursing home). The cost-evaluation questionnaire will be returned with the falls calendar at three, six, nine, and twelve months after the first visit to the research outpatient clinic. In case no calendar sheet or questionnaire is received, or when it is completed incorrectly, the calendar sheet or questionnaire will be completed by telephone.

During the last visit to the outpatient clinic, one year after the first visit, all physical performance tests are conducted, as well as questionnaires regarding medical history, drug use, quality of life, and fall risk profile. Adherence to the drug-use recommendations (complete withdrawal, lowering of dosage, or substitution) will be evaluated by reassessment of drug use as described above. Information of the participants regarding medical history and drug use will be verified by the General Practitioner and local pharmacist.

### Sample size calculation

A total number of 620 patients will be included in the study, 310 in the control group and 310 in the intervention group. Calculation of the required sample size is based on the assumption that the annual cumulative incidence of further falling is 50% without intervention [40], a 15% drop-out (including death) [11], drug withdrawal being possible in 50% of the participants in the intervention group and a 50% decrease of further falls among participants with successful withdrawal [18]. A single-sided test with an alpha level of 0.05 and a beta of 0.2 indicates that 310 patients in both groups is sufficient in order to detect a 25% decrease of respondents reporting further falls in the intervention group.

### Statistical analysis

Data will be primarily analyzed according to the intention-to-treat principle. Patients with protocol violations will be followed up, and data will be recorded. Data will be analyzed with and without inclusion of patients with protocol violation. At baseline, differences in baseline characteristics will be compared between the intervention and control group in order to assess comparability between the two groups. Student's T-test (parametric numeric data), Mann-Whitney U-test (nonparametric numeric data) or Chi-square test (categorical data). Data will be presented as mean ± SD (parametric data) or medians and percentiles (non-parametric data).

The hazard ratio for falling will be calculated using a Cox-regression model. Herein, the time between the intervention (*i.e*., drug assessment/change or not) and the first and/or second fall will serve as the primary outcome measure. Fallers will be defined as those who will fall once or more during the one-year follow-up. Differences in cumulative incidence of falls will be analyzed using log-linear or Poisson regression, adjusted for over dispersion because of interdependence among the dependent variable (falls). Differences in adverse health effects between both trial arms will be assessed using Chi^2 ^testing. Several subgroups will be distinguished in order to examine whether the effect of the intervention depends upon sex, age, race and risk of future falls. Since healthcare costs per patient are typically highly skewed, non-parametric techniques will be used to derive a 95% confidence interval for the differences in distributions of the costs. In a sensitivity analysis the impact on cost-effectiveness of statistical uncertainty on the main study outcomes will be determined (uni- and multi-variable).

The association between genetic polymorphisms and falls history will be evaluated using a multivariate logistic regression analysis. A *p*-value of < 0.05 will be used as threshold for statistical significance.

### Ethical considerations

The study will be conducted according to the principles of the Declaration of Helsinki (59^th ^World Medical Association General Assembly, Seoul, October 2008 [41]) and in accordance with the Medical Research Involving Human Subjects Act (WMO). The Medical Ethics review board of the Erasmus MC acts as central ethics committee for this trial (reference number MEC-2008-254; NTR1593). In addition approval has been obtained from the local Medical Ethics review boards in all participating hospitals. An information letter regarding the patients' participation and severe abnormal findings will be sent to their general practitioners, unless a patient does not agree with this. Liability insurance has been obtained, which is in accordance with the legal requirements in the Netherlands (Article 7 WMO and the Measure regarding Compulsory Insurance for Clinical Research in Humans of 23th 2003). This insurance provides cover in case of damage to research subjects through injury or death caused by the study.

## Discussion

The strength of this study is that a single intervention, the withdrawal of fall-risk increasing drugs, will be studied versus 'usual care' using a randomized controlled approach. The study results will provide valuable knowledge for clinicians and healthcare policymakers on the necessity of withdrawal of fall-risk increasing drugs in falls prevention strategies in the older population. If proven effective and cost-effective, fall-risk increasing drugs withdrawal in persons with a high risk of recurrent falling, might lower the risk of future falls and consequently contribute to reductions in fall-related injuries, related healthcare consumption, and costs. As far as we are aware, up till now no large RCT's have been published reporting the effects of withdrawal, dose reduction or substitution of fall-risk increasing drugs after a fall. The inclusion of patients started October 2008 and is expected to be complete by July 2011. Because of the one-year follow-up period, presentation of data can be expected in the second half of 2012.

## Competing interests

The authors declare that they have no competing interests.

## Authors' contributions

KAH, NVDV, EMMVL, OJDV, EFVB, PL, PP and TJMVDC developed the trial and drafter the manuscript. TJMVDC will act as trial principal investigator. SP and EFVB assisted in the design of the healthcare consumption questionnaire and will perform the health economic analyses. KH, NB, EMMVL, FUSM, AGU, EFVB and NVDV will perform statistical analysis of the trial data. KAH, NVDV, OJDV, NB, AJHK, GZ, MMMB, MRDV, FUSM, PL, PP, and TJMVDC will participate in patient inclusion and assessment. All authors have read and approved the final manuscript.

## Pre-publication history

The pre-publication history for this paper can be accessed here:

http://www.biomedcentral.com/1471-2318/11/48/prepub
